# The mothering experience of women with FGM/C raising ‘uncut’ daughters, in Ivory Coast and in Canada

**DOI:** 10.1186/s12978-017-0309-2

**Published:** 2017-04-05

**Authors:** Sophia Koukoui, Ghayda Hassan, Jaswant Guzder

**Affiliations:** 1 Psychiatry Department, Transcultural Research and Intervention Team, McGill University, 7085 Hutchison, room 204.2.1, Montreal, QC H3N 1Y9 Canada; 2 Department of Psychology, University of Quebec at Montreal, Montreal, QC Canada; 3 Transcultural Research and Intervention Team, C.P. 8888 Succ. Centre-Ville. DS- 4797, Montréal, QC H3C3P8 Canada; 4 McGill Faculty of Medicine, Department of Psychiatry, Center for Child Development and Mental Health, Institute of Community and Family Psychiatry, 4335 Cote St Catherine Rd., Montreal, QC H3T 1E4 Canada

**Keywords:** Female genital mutilation/cutting, Qualitative research, Mothers, Migration, Abandonment of FGM/C

## Abstract

**Background:**

While Female Genital Cutting (FGM/C) is a deeply entrenched cultural practice, there is now mounting evidence for a gradual decline in prevalence in a number of geographical areas in Africa and following migration to non-practicing countries. Consequently, there is now a growing number of women with FGM/C who are raising ‘uncut’ daughters. This study used a qualitative methodology to investigate the experience of women with FGM/C raising daughters who have not been subjected to the ritual. The aim of this study was to shed light on mothers’ perception of the meaning and cultural significance of the practice and to gain insight into their mothering experience of ‘uncut’ girls.

**Methods:**

To this end, in-depth interviews were conducted with fifteen mothers living in Abidjan, Ivory Coast and in Montreal, Canada (8 and 7, respectively).

**Results:**

Thirteen mothers intrinsically refused to perpetuate FGM/C onto their daughters and two diasporic mothers were in favour of FGM/C but forewent the practice for fear of legal repercussions. Whether the eschewing of FGM/C was deliberate or legally imposed, raising ‘uncut’ daughters had significant consequences in terms of women’s mothering experiences. Mothers faced specific challenges pertaining to community and family pressure to have daughters undergo FGM/C, and expressed concerns regarding their daughters’ sexuality. Conversely, women’s narratives were also infused with pride and hope for their daughters, and revealed an accrued dialogue between the mother-daughter dyad about cultural norms and sexuality. Interestingly, women’s mothering experience was also bolstered by the existence of informal networks of support between mothers with FGM/C whose daughters were ‘uncut’. These communities of mothers engaged in open dialogue about the consequences of FGM/C and offered reciprocal solidarity and support in their decision to forego FGM/C for their children.

**Conclusion:**

Women with FGM/C who are raising ‘uncut’ daughters in their homeland and in their country of immigration vastly report a positive experience. However, they also face specific challenges related to immigration, psychosocial, and psychosexual considerations, which must be tackled from a multidisciplinary perspective.

## Plain English Summary

Female Genital Cutting (FGM/C) consists in the removal or alteration to the female external genital area for non-medical reasons. It is a deeply entrenched cultural practice and while the prevalence rate is elevated, there is now a gradual decline in most practicing countries. At the same time, global migration makes it such that women with FGM/C are living in countries that do no perform the practice. Vastly, they do not tend to perpetuate FGM/C post-migration. Hence, at the global level, there is now a significant number of women with FGM/C raising ‘uncut’ daughters. The main purpose of this research is to better understand their mothering experience.

To this end, in-depth interviews were conducted with fifteen mothers living in Abidjan, Ivory Coast (7) and in Montreal, Canada (8).

Raising ‘uncut’ daughters had significant consequences: mothers faced specific challenges pertaining to community and family pressure to have daughters undergo FGM/C, and expressed concerns regarding their daughters’ sexuality. Concomitantly, they also expressed pride and hope for their daughters, and engaged in conversations with them about cultural norms and sexuality. Interestingly, their mothering experience was bolstered by informal support networks of mothers with FGM/C raising ‘uncut’ daughters. These communities of mothers offered support in their decision to forego the practice for their children.

In conclusion, women with FGM/C raising ‘uncut’ daughters in their homeland and in their country of immigration vastly reported a positive mothering experience. However, they also face specific psychosocial, psychosexual and policy issues, which must be tackled from a multidisciplinary perspective.

## Background

Female Genital Cutting (FGM/C) encompasses a number of practices, which consist in the removal or the injury to the external female genital area [1]. It is estimated that 200 million women alive today have undergone FGM/C [2] and that approximately 3 million girls are at risk for the practice on a yearly basis [2, 3]. The World Health Organization (WHO) has established a classification system that categorizes the various forms of FGM/C [4]. Type I, often referred to as clitoridectomy, consists in the partial or total ablation of the clitoris. Type II, termed excision, corresponds to the removal of the clitoris and part or all of the labia minora. Type III, or infibulation, consists of the cutting of the clitoris, labia minora and majora, followed by stitching of the vulvar area, which leads to a narrowing of the vaginal opening. Type IV involves alternate practices, which do not fall into the three aforementioned categories. In recent years, subcategories have been added to better account for the variations in the procedure [5]. A significant body of literature has reported immediate, short and long-term consequences, such as severe pain, haemorrhage, acute urinary retention, septicaemia, anaemia resulting from blood loss, recurrent urinary tract infections and vulvar ulcers [1, 6–11]. Remote complications include birthing difficulties and adverse obstetric outcome, which are more deleterious according to the extent of the resection. Such risks include obstetric fistula resulting from obstructed or prolonged labour, postpartum haemorrhage, and neonatal death [12, 13]. While there are unarguably biomedical consequences to the practice, notably for type III FGM/C, it should be noted that sensationalistic accounts have also been purported regarding the potential sequelae, possibly as a deterrent from the practice [14, 15].

FGM/C is a deeply entrenched cultural procedure, which is part of the cultural heritage of a wide number of ethnic groups. Research has underscored the importance of FGM/C in consolidating communal ties, affiliation and a shared group identity. The notion of purity, sexual property and honour are central to the rhetoric surrounding the practice [16, 17]. While the significance of FGM/C varies amongst different ethnicities, historically and culturally, the practice has often served the instrumental function of regulating interactions between the sexes, of cultivating values of sexual modesty, as well as forging community and family ties [18, 19]. These motives are most significant in the context of childrearing, particularly when daughters, unlike their mothers, do not bear this cardinal cultural marker of their group of origin.

While rates of FGM/C remain elevated in most practicing countries, some showing a fixed or slightly increased prevalence rate in spite of global health programs and campaigns, studies point to a gradual decline in prevalence and endorsement in a number of geographical areas and studies suggest that in a number of African countries, fewer daughters than mothers have undergone FGM/C [20–23]. Culture is ever evolving and dynamic, and as stated by Berg and Denison, FGM/C is indeed ‘a tradition in transition’ [16]. Consequently, an increased number of “cut” mothers living in Africa are raising daughters who have not undergone the procedure. Concurrently, global migration makes it such that more African women with FGM/C are migrating to geographical spaces that do not practice the ritual and which overall, have been more stringent in applying anti-FGM/C legislation and criminalization than African countries [24, 25]. While accurate data on FGM/C prevalence is difficult to obtain, there appears to be a general tendency to forgo FGM/C post-settlement [26]. Moreover, a number of qualitative studies over the past decade indicate that immigrant communities from practicing countries now have a tendency to hold negative views of the procedure and to forgo FGM/C. Reports of a shift in attitude and behaviour toward FGM/C abound from Canada, Norway, Sweden, Britain, Israel [27–33]. It should be noted however that in such qualitative studies, a physical examination of the daughters to ascertain their “cut/uncut” status is not performed, as the methodology and potential outcomes would be fraught with ethical and legal entanglements. Hence, there is a presumption of good faith and transparency inherent to studies reporting that parents are mostly disinclined to perpetuate FGM/C post-migration. Taken together, these demographic data and qualitative research results suggest that most diasporic mothers with FGM/C are raising daughters who do not bear this cultural marker.

While the experience of motherhood is dependent on individual, transgenerational and collective histories, it is also coloured by contemporary views on cultural practices and external influences, notably in an increasingly globalized world. The literature on diasporic communities’ stance on FGM/C is still at its inception, however it appears that beyond a behavioural change based on fear of legal sanction, there is a shift in attitude toward FGM/C in diasporic communities. As mentioned by Gele, one may posit that some of the social forces influencing the continuation of the practice are attenuated through immersion in a society that does not attach social status to the practice [27].

While studies point to a gradual abatement of FGM/C in several African countries and a general tendency for diasporic communities to forego the practice, no study to date has investigated the experience of women with FGM/C who are raising ‘uncut’ daughters. The present qualitative study aims to bridge this gap by shedding light on women with FGM/C’s experience of raising ‘uncut’ daughters, both in Ivory Coast and in Canada.

## Methods

### Description of participants

The present qualitative study was conducted among fifteen African mothers who underwent FGM/C and were raising “uncut” daughters. Eight women resided in an urban setting in Ivory Coast and seven lived in Montreal, Canada. The selection criteria were for women to have undergone FGM/C, to have at least one daughter who did not undergo the practice, and to be over 18 years of age. All women who came forth to share their narrative were included in the study, insofar as they fit the selection criteria.

The mothers residing in Montreal, Canada, were between the ages of 36 and 59. They originated from the horn of Africa (Somalia, Djibouti and Ethiopia), West Africa (Mali and Guinea) and Egypt. The participants were either Christian or Muslim. All were born and raised in Africa, and arrived to Canada well into adulthood. The motives given for immigration were to seek a more favourable quality of life for themselves and their family; to flee the civil war in Somalia; two mothers migrated specifically to prevent FGM/C for their daughters; and one participant traveled to Canada to seek fertility treatment, and the couple opted to stay after the child’s birth.

Women in Ivory Coast were between the ages of 28 and 62. Seven women were originally from Ivory Coast, and one was from Burkina Faso. All were born and raised in the Western part of Ivory Coast (in the districts of the Eighteen Mountains and Cavally) and resided in an urban setting (in Abidjan or a nearby city). The mothers were all either Christian or Muslim. A summary of sociodemographic information and reasons for eschewing FGM/C is provided in Table 1.

**Table 1:** Description of the participants and motives for discontinuation of FGM/C for their daughters

Name	Country of residence	Age at FGM/C	Country of origin	Religion	Number of daughters	Reported motive(s) for not having daughters undergo FGM/C
Aicha	Canada	5–6 years old	Somalia	Muslim	1	1- Not mentioned in the Quran. Not mandated by Islam.
Bilal	Canada	80 days	Ethiopia	Christian	1	1-Does not want her daughter to suffer the consequences of FGM/C that she is enduring: a) intimacy is unsatisfactory, b) feeling of not being whole as a woman.
Hanan	Canada	7–9 years old	Djibouti	Muslim	3	1- Does not want daughters to suffer the consequences of the practice (participant had a severe haemorrhage requiring urgent hospitalization).
Fatou	Canada	6–7 years old	Guinea	Muslim	2	1- Suffered too much from the procedure and refuses to have her daughters undergo FGM/C.
Fatma	Canada	7 years old	Egypt	Muslim	3	1- Husband does not want daughters to undergo FGM/C.
						2- Paediatrician advised her not to have FGM/C performed.
						3- Friends from the diasporic Egyptian community are against FGM/C and did not do it for their daughters.
Binta	Canada	6 years old	Guinea	Muslim	1	1- Suffered deeply from the practice and refuses to have her daughter experience the pain of FGM/C.
Mariam	Canada	2 months old	Mali	Muslim	1	1- Pediatrician advised her not to have daughter undergo FGM/C.
						2- Afraid that her daughter will be taken away by Youth Protection if her daughter has FGM/C.
Helen	Ivory Coast	18 years old	Ivory Coast	Christian	2	1- Suffered physically from the procedure (fainted) and is concerned about the sexual consequences of FGM/C. Does not want her daughters to undergo the ritual.
Amina	Ivory Coast	10 years old	I Ivory Coast	Muslim	3	1- Does not want her daughters and grandchild to suffer as she did. Perceives FGM/C as an antiquated practice.
Alice	Ivory Coast	10 years old	Ivory Coast	Christian	1	1- Does not want her daughter to suffer as she did and currently does (recurrent infections and haunted by the memory of the event).
						2- Finds that FGM/C makes delivery more painful.
						3- Believes men nowadays prefer to be with “uncut” women, and so it would be detrimental to daughter to have her undergo FGM/C.
Martine	Ivory Coast	14 years old	Ivory Coast	Christian	1	1- Suffered from the procedure (fainted) and does not want daughter to suffer as well.
						2- Believes men nowadays prefer to be with “uncut” women, and so it would be detrimental to daughter to have her undergo FGM/C.
Awa	Ivory Coast	16 years old	Ivory Coast	Muslim	3	1- Her husband left her due to the consequences of FGM/C (sexuality was an issue). She does not want her daughter to have conjugal difficulties.
Samira	Ivory Coast	5 years old	Burkina Faso	Christian	2	1- Believes FGM/C to be a harmful practice.
Amina	Ivory Coast	4 years old	Ivory Coast	Muslim	1	1- Suffered from the practice and almost passed away (from haemorrhage). Believes the practice is harmful and highly dangerous. Does not want daughter to undergo the same experience.
Rokhaya	Ivory Coast	5–6 years old	Ivory Coast	Christian	2	1- Suffered from the practice and knows of a child who passed away after undergoing FGM/C. Did not want to subject her daughters to the ritual.

### Data collection

The data collection was conducted both in Canada and in Ivory Coast. Women in Canada were recruited through key members of the diasporic African communites and through the snowball technique. The participants in Ivory Coast were initially recruited through two NGOs (ONEF — Organisation Nationale pour la Femme, l’Enfant et la Famille, and NGO Solidarity) based in Abidjan. These organizations carry out prevention interventions within the community and are lead by Ivorian women. While NGO workers informed the participants of the study, the latter were not recipients of services provided by these organizations. In addition, two women came forth to be enlisted in the study following referral from acquaintances who had participated in the research project.

Women were handed an information sheet stipulating the aim of the research project and the main themes of the study. A consent form addressed issues of anonymity and the right to decline participation prior, during and following their participation. They completed a sociodemographic questionnaire including date of birth, country of origin, country of residence, ethnicity, marital status, as well as the number and the gender of their children. The completion of the sociodemographic questionnaire was followed by semi-structured interviews conducted by the first author. All interviews were audiotaped and transcribed verbatim. Three women in Ivory Coast expressed a preference for a small-group interview, and this was respected. This data collection method has proven effective in previous studies on FGM/C [27, 34] and it is congruent with local culture, which favours group discussions between women [27, 35]. Refreshments and light food were offered to the participants, and travel expenses were reimbursed. Women were informed of the goal of the study and methods of data collection. They were informed of their right to refuse to respond to any question and to withdraw from the study at any time without prejudice. The participants were provided with professional references in case they were to experience any kind of distress following the interview. Women in Canada were referred to a psychologist who specializes in transcultural clinical work, and women in Ivory Coast were referred to two psychosocial workers who provide support to women with FGM/C. Written consent was sought prior to any data gathering. Each participant was given a code to safeguard anonymity. This research project was approved by the Université du Québec à Montréal Psychology Department Ethics Committee.

### Analysis

A qualitative analysis of research data was performed to gain insight into the perception of FGM/C and the mothering experiences of the participants. Given the aim of the study, a qualitative approach was best suited, as it affords a better understanding of the complexities and subtleties of women’s experiences in raising “uncut” daughters [36]. The semi-structured interview guide focused on specific themes, namely perceived motives for the practice of FGM/C, personal experience of FGM/C, transition to motherhood, the decision to forego FGM/C for daughters, maternal experience of raising ‘uncut’ daughters, and family dynamics. A thematic analysis was applied, based on L’Ecuyer’s mixed categorisation model [37]. Assiduous consideration was paid to the distinctiveness of women’s mothering experiences, as well as the ways in which they coalesced. Women’s narratives were analysed extensively, and a horizontal analysis served to identify the similarities and differences in their experiences. The interview verbatim were analyzed by the principal investigator and discussed between the authors for consensus with regards to themes and categories.

## Results

### Mothering experience: challenges and concerns

Most women in our sample expressed pride and relief that their daughters did not undergo FGM/C. However, their mothering experience was still tainted by several challenges, which are addressed in the following section.

#### Pressure from the extended family network

A number of participants declared that FGM/C is a *conditio sine qua none* to a woman’s marriage. They asserted that beyond a union between two individuals, marriage is also an alliance between families, and it is incumbent upon parents to ensure their daughter’s marriageability by keeping tradition. This significantly complicated parental objection to FGM/C, because of the socioeconomic disadvantage and social vulnerability inherent to a woman’s celibacy. Such was the experience of several participants, whose own parents later expressed their disproval of the practice. They had felt coerced to endorse the group law in order for the family to be part of the social body. Most participants stated that, in their locality, ‘uncut’ daughters and their family are typically shunned from the community and are prevented from partaking in traditional ceremonies and specific group gatherings. The narrative below from an Ethiopian mother, Bilal, offers an illustration of the social ramifications of foregoing FGM/C for the entire family network and the community:

“The perception they have back home, if the girls are not circumcised, they will be very active sexually, and they will lose their virginity. And that’s a shame. Back home if you don’t have your virginity when you are married, that’s a shame to you, that’s a shame to your family, a shame to your neighbour, a shame to everybody!”

This narrative underscores the importance of communal ties, affiliation and a shared group identity. It is congruent with the fact that the foremost difficulty disclosed by mothers was pressure and involvement of the extended family network, which frequently inquired whether their daughters had undergone FGM/C. This family involvement stemmed from aunts as well as female elders from the paternal side of the family. Several participants in Abidjan (25%) had repeatedly warned their entourage that they did not want their daughters to be “cut” and complained about what they described as an antiquated intrusion on their maternal purview. Mothers living in urban settings in Ivory Coast mentioned that their daughters were better protected in the city, and several refrained from taking them to their home village for fear that they would be subjected to FGM/C. Diasporic mothers (43%) also reported being pressured by relatives in their home country. They expressed a lack of safety in their homeland, where community involvement was ineluctable. Consequently, several opted not to take their daughters on vacation to Africa in order to avert the ritual. Such was the experience of Binta, a Guinean mother residing in Canada:

“I’ve seen a lot of cases like that. It is not the parents who do it, but it’s like, you’re living in an environment that is not safe for girls. It’s unsafe. It’s really unsafe. If they realize that the child was not circumcised, they will come and take her to do it. And what are you going to do after that? Take them to Court? And it is not just members of your family or your husband’s family. I am telling you, it’s anybody. As long as there is even a small link between you and them, they’ll do it. (…) Each time I talk on the telephone with one of my husband’s aunties she says ‘your daughter is still in Canada. It hasn’t yet been cut’. One of them wants to do it, because she’s a circumciser (…). She says ‘the day your daughter comes home, I will do it’. So I’m not taking any chances. My daughter is not going home, I’m not going to take that risk.”

Women’s narratives indicate a tendency to avoid geographical spaces where FGM/C is still widely pervasive for fear that their daughters might be exposed to the practice without their consent. Maternal protection superseded respect for their elders’ preference regarding FGM/C, which for some mothers, came at the cost of geographical dislocation and erosion of family ties.

#### Concerns about ‘uncut’ daughters’ burgeoning sexuality

In spite of the vicarious satisfaction expressed by most participants, the shift was not without ambivalence for a Malian and an Egyptian diasporic mothers. Of note, both mothers were amongst the three participants in our sample who had no conscious recollection of their own FGM/C experience. The Malian woman was ‘cut’ shortly after birth, and the Egyptian participant underwent the procedure in a hospital setting, under anaesthesia and sedation. Both opted not to have their daughters undergo FGM/C for fear of legal repercussions in Canada. Their daughters’ paediatrician had addressed FGM/C and deterred them from carrying on the practice. Mariam was an articulate Malian woman in her early forties. She was quite traditional, and conservative in her views. Elements of her culture of origin permeated her discourse and were central to the way in which she organized her life. For this mother, her child’s ‘uncut’ body was cause for curiosity and anguish:

« My daughter, she’s a teenager now. She’s starting to tell me ‘mom, my friends have boyfriends you know’. (…) In my time, at 15, we didn’t even think about boys, and we feared our parents. But this is not the same generation. So that’s what I’m afraid of. And above all, I tell myself that my daughter wasn’t circumcised. According to what we hear, people who are circumcised and those who were not, it’s not the same thing. Those who were circumcised can wait. But it’s the opposite for those who weren’t circumcised. So that’s what I have in mind when it comes to my daughter, and I won’t stop talking to her. She knows that to us, some things are sacred. ».

Miriam perceived the libido of ‘cut’ and ‘uncut’ women as being antithetical, stating that “*it is the opposite for those who were not circumcised*”. Now an ‘uncut’ teenager, her daughter’s burgeoning sexuality was cause for maternal concerns.

With regard to adolescence and sexual temperance still, the perception of Fatma, the Egyptian mother of three, offers an illustration of FGM/C’s speculated ability to prevent premarital relations. She mentioned that currently, the prevalence of FGM/C is plummeting in her country of origin, except illegally in remote villages. Although, according to her, the practice was beginning to fall into obsolescence, she was not opposed to its continuation:

« Here in Canada it must be cut off! It protects and prevents girls from going out with boys. She’s going to go out and only stay with her friends, it’s better! I’m in favour of that. I’m against the fact that here, they’re really in a hurry! And it’s not clean on both sides. So they should do it in Canada, because I see that here, young people are very eager. Over there [Egypt] the youth are with their family at home. But here, some parents let their daughter go anywhere! It’s not good. So it’s better to do it here than over there”.

For Fatma, FGM/C was of little use in Egypt, where daughters are protected within the confines of the family nest and the collective enforcement of social rules and gender roles. But far from deflecting its relevance overseas, she propounded that FGM/C would serve greater purpose if ‘exported’ to Canada —where her daughters were being raised— as an impediment to youth’s sexual licence. Hence, for several participants, mothering ‘uncut’ daughters raised significant concerns about transmitting cultural values of sexual modesty.

#### Migration policies

A diasporic woman from Guinea recounted a painful experience with regard to the protection of her daughters. After the birth of her first daughter, she made the resolution to leave the country to prevent her from undergoing FGM/C. She was a highly educated woman, who was prosperous and financially independent in her home country. She had not anticipated how complex and lengthy the migratory process would be, but remained firm and resolute in her intentions. While undergoing the immigration process to Canada, she became pregnant with her second child: also a daughter. The participant eventually received immigration papers, but because the initial request was formulated after she had given birth to only one child, she was not granted papers for all her children. She left the country with one child, under the assumption that following migration, the process of having the second daughter come to the country would be a formality. Unfortunately, such was not the case. Her second daughter immigrated years later, after undergoing FGM/C. This mother recounted with great sadness the painful experience of initiating the migration process to spare her daughter the pain of FGM/C, only to leave the other daughter behind. Her guilt was compounded by her second child verbalizing feelings of abandonment, anger and despair that she had not been spared ‘like her sister’. Our participant had attempted several times to inquire about her experience, but her daughter refused to address FGM/C, stating that she did not want to be reminded of what happened while her mother was away. The youngest daughter had migrated one year before the interview was conducted. Needless to say, the reunification process was arduous. Mother and daughters were in family therapy and slowly repairing and cultivating family ties and trust, which had been broken in part by policies, customs and distance.

### Mothering experience: individual and collective successes

The following section addresses the many positive facets of mothering ‘uncut’ daughters, as described by the women in our sample.

#### Mother-daughter relationship: on maternal empowerment and vicarious protection

With the sole exception of the two diasporic mothers whose experience was detailed above, all women expressed relief and gratitude for the opportunity to raise their daughters in an environment that permitted them to demur the practice. Their decision was both endorsed within their novel social setting and buttressed by law. They made such statements as “*thank goodness we have moved to the city so I don’t have to worry about that*”, “*at least I know my daughter is safe here*”. Over the course of the interviews, women reflected upon their own painful experience of FGM/C. This incarnate, intimate knowledge catalyzed their deep-seated rejection of FGM/C for their children. The following excerpts illustrate their vehement disapproval of the ritual and their unwavering commitment to protect their daughters:

Alice: “I regret it [undergoing FGM/C] so much! So much! If someone came to me now telling me ‘I’ll give you billions of dollars if you circumcise your daughter’, I can’t! I can’t! I can’t because of the pain that I know today (…). As long as I’m alive, no one will touch my daughter!”.

Awa: “As I am sitting in front of you, maybe if I were dead it would be another story. But as long as my two eyes are open, my daughters, never! My daughters will never, never do that! We did not know what it was about and we fell into that trap. I was fooled once, but I won’t be fooled again (…) All I know today is that we’re going to fight for our daughters”.

A sense of agency and maternal protection exuded from women’s discourse. The vicarious protection they were able to afford their offspring constituted a source of relief and gratification that acted as a counterpoint to their own memories and pain. Mothers were appreciative of the opportunity to raise their daughters in an environment that offered protection against FGM/C, and took solace in knowing that their daughters would know another destiny.

#### A new mode of transmission of cultural ethos

The participants’ narratives indicate that the upholding of moral propriety and sexual temperance are central motives to FGM/C. They often stated that these were core values of their culture of origin in terms of women’s expected behaviour. Hence, repudiating FGM/C triggered a wave of questioning as to how they would transmit these values to their daughters without resorting to the ritual cutting, particularly in light of their displacement to a setting that enabled more casual interactions between genders. It was a fundamental concern for the two diasporic mothers who were in favour of FGM/C, but also for mothers in our sample who rescinded the practice. Maternal angst and curiosity about their daughters’ sexuality emanated from their discourse. Foregoing FGM/C caused them disquiet about their daughters’ libido. More specifically, they feared that their daughters would not be able to refrain from engaging in premarital sexual relations, and hence not be virgins upon marriage. They also feared that their sexual drive would lead them to have extramarital affairs. These concerns highlight their wish or preference regarding their daughters’ sexual behaviours, but it also underscores a focus on marriage and family ties, which could be halted or compromised by premarital and extramarital sexual relations. In order to curtail sexual “promiscuity” without resorting to FGM/C, several participants both in Ivory Coast and Canada engaged in discussions with their daughters around sexuality. Several women explained that in light of the novel sociocultural environment in which they were immersed, they would have regular conversations with their daughters about relationships and sexuality. Several spoke to the importance of forging dialogue with daughters and asking them questions, unlike “*in the old days*”. Hence, they adapted their childrearing practices by prompting open discussions about coming of age, reproductive health and sexuality.

#### The emergence of communities of mothers against FGM/C

All women in our study mentioned the taboo nature of FGM/C in their community of origin. Many had felt compelled to raise the issue with their mother following their own ritual cutting, but were quelled by elders who admonished them to stay silent about the practice. Several participants expressed a protracted yearning for answers to the questions they had generated in their child’s mind; questions which were still lingering in the backdrop. Now well into adulthood, a number of women reported breaking this taboo and engaging in a dialogue about FGM/C with other mothers in their community, who had also undergone the practice. Women residing in Ivory Coast and in Canada reported this phenomenon of support through dialogue. Conversations centered around the consequences of FGM/C in different facets of their lives and on their commitment to protect their daughters. Women recounted positive feelings regarding these discussions and felt a sense of mutual understanding, solidarity, and a collective engagement to shield their daughters from the ritual. These organically formed circles of women consisted of sisters, in-laws, fellow Church attendees, and women who met at information sessions organized by ONEF-NGO and pursued conversation outside the realm of the organization. Both Christian and Muslim mothers reported engaging in such discussions, irrespective of the geographical location, as women residing in Ivory Coast and Canada reported this communal experience.

## Discussion

To this day, the anchoring of FGM/C as a cultural practice remains considerable in our participants’ countries of origin [2]. However, both globalization and the continued commitment of African women and the international community to obliterate the practice have spurred regional declines in FGM/C. To the best of our knowledge, this is the first research study investigating the experience of women with FGM/C raising daughters who have not undergone the practice.

For the two diasporic mothers in favour of FGM/C, the endorsement of traditional values with regards to sexuality was mostly rooted in maternal concerns over raising their daughters in a foreign land. There was hence a shift in meaning of FGM/C brought about by migration: post-migration, the practice operationalized the transmission of a cultural ethos of sexual modesty, in a foreign land characterized by a more licentious context and different child-rearing practices. A recent study conducted with Somali women in Oslo echoes similar concerns, as mothers were preoccupied by the impact of their daughter’s ‘uncut’ status on their burgeoning sexuality [28]. A study exploring Nigerian mothers’ perceptions of FGM/C also reveal such concerns, as 44.2% of mothers believed that foregoing FGM/C irrevocably led to promiscuity [38]. For women with FGM/C raising ‘uncut’ daughters, the challenges inherent to raising an adolescent girl are compounded by the difficulty of mothering a daughter that is not quite like themselves. Unlike immigrant women from countries that do not practice FGM/C, they face the additional challenge of raising a daughter that is different from them in a central locus of their womanhood. The identification and projection onto their daughters is rendered more complex by their physical ‘otherness’ and its implications in terms of potential pleasure and sexual experiences.

The mothers in our study reported that young girls who avert FGM/C are a source of shame to the entire community and are forbidden from partaking in social gatherings and ceremonies. This significantly complicates the eschewing of FGM/C, because of the socioeconomic precariousness and social vulnerability inherent to a woman’s celibacy. Our findings echo the results of an Ethiopian study, where social acceptance was mothers’ prime reported motive for carrying out FGM/C (90%) [39]. Likewise, women in the study also vastly reported an involvement of elderly women to perpetuate the rite of passage onto their daughters (74.4%).

While displacement to urban settings in Ivory Coast and to Canada attenuated the impact of stigmatization in daily life, it failed to curtail familial pressure. Indeed, mothers in Ivory Coast (25%) and in Canada (43%) reported being berated by the larger family system to perform FGM/C.

Because it is incumbent upon mothers to ascertain their daughters’ FGM/C, their stance was considered a desecration of communal rules.

### The underpinnings of mothers’ decisions to forgo FGM/C in the context of the African social matrix

The extended family’s continued pressure for the perpetuation of FGM/C indicates that collective involvement in maternal decisions tends to still be part of contemporary social dynamics. It attests to the organization of the social fabric, which is characterized by the interconnectedness of Self in a significant number of African ethnicities. While each family and entourage have their own dynamics, hierarchy and ways of engaging with the collective, the notion of a Person tends to be highly relational in many FGM/C-practicing ethnicities — one’s identity and personhood are often tied to that of their family, and extends to the social group [40, 41]. This communal mode of being into the world seeps into the realm of sexuality. As Wangila aptly argues, “sexuality is not only understood in relation to an individual’s marital relations but in relation to the role of marriage, social status, spiritual matters, and social ideologies in the maintenance of a given community” [42]. This interdependence and interconnectedness bolsters cohesion and support within the social matrix, but creates greater pressure for conformity as well, which mothers experienced in light of their decision to forgo FGM/C.

Our results indicate that the geographical insulation brought about by displacement did not shield diasporic mothers from matriarchal pressure. Our finding that pressure emanated exclusively from female elders is in line with previous research studies pointing to women’s involvement in the practice [43, 44], and must be interpreted in light of the concept of Motherhood in our participants’ cultures. Far from being confined to the realm of strict lineage, motherhood is also a social role and responsibility where women are collectively involved in the raising of a child. They are also granted the role of keepers of tradition and are accountable for the transmission of moral and cultural values to their offspring [27, 45]. As such, failure to uphold traditional practices such as FGM/C is apprehended as maternal negligence on part of the entire collective of women who are accountable for the daughter’s upbringing. In addition, decision-making power is often hierarchically distributed within the family system, with Mother Elders relishing a greater social status. As such, their position on the family map confers them with greater influence and power [46, 47]. Such a status has the double consequence of legitimizing the involvement of Mother Elders in the FGM/C status of young girls, and granting appreciable weight to their insistence on the perpetuation of FGM/C. Hence, one must not underestimate the boldness of younger mothers who forgo the practice in spite of matriarchal pressure.

The women in this study were facing a mothering context that was unprecedented in their group of origin: that of raising daughters that had not experienced a cornerstone of their culture of origin. Traditionally in communities that practice FGM/C, Mother Elders counsel younger mothers in caring for their children. However, our participants could not seek information from Mothers Elders’ experience, as this was a novel mothering context. Neither these younger mothers nor their elders had insight into the incarnated bodily experiences of “uncut” daughters. As our data suggests, several mothers are now prompting a more open dialogue with their ‘uncut’ daughters regarding sexuality.

### Circles of mothers rejecting FGM/C: communal healing and solidarity

The creation of circles of solidarity between women who underwent the practice and oppose its perpetuation reveals an organic mode of healing. Indeed, while their ritual cutting had been surrounded by a shroud of secrecy leaving many questions unanswered, dialogic circles enabled women to break the silence on their shared incarnate experience of FGM/C and its multifaceted contemporary reverberations. Furthermore, as children, our participants were not interrogated about their volition to undergo the ritual. Yet feelings of pride and determination permeated their discourse regarding their collective engagement for the protection of their daughters. We hence posit that in contrast to their experience as children subjected to the practice, their collective stance against FGM/C and resolute commitment to protect their daughters operationalized a radical shift towards a position of agency and a sense of assertiveness. It may also be that these circles held special meaning from a dialectic of inclusion versus exclusion. Several women, whether they resided in urban settings in Ivory Coast or in Canada, alluded to feelings of ostracism due to their eschewing of FGM/C. The novel bonds with a community of women and mothers who resist and fight against the practice fostered a reconfiguration of their social network. Such a network is often crucial to migrant mothers who experience isolation as they navigate through multiple cultural systems, which may be conflicting with regards to mothering practices [48].

### Limits and perspectives

This study has several limitations. The fact that most Ivorian women were recruited through two NGOs with a clear positioning against the practice creates a sample bias. However, several participants spontaneously reported some advantages to the practice (notably, a ‘reasonable’ libido and greater cleanliness), indicating that the mode of recruitment did not preclude them from delineating nuances in their perceptions and experience.

This study did not take into account FGM/C typology and categorize women according to the extent of the resection. The focus here was on the experience of women who have undergone the ritual, mothering daughters who did not. Furthermore, the limited reliability of self-reported forms of FGM/C compromises the accuracy of such data [49].

Because participants originated from several ethnicities, the results are not generalizable to one specific group. However, the goal of the study was not to seek generalizability with regard to a specific ethnic group, but rather to shed light on the experience of women with FGM/C who were raising daughters whom did not bear this cultural marker of their group of origin. Moreover, geographical location and a shared ethnicity are no guarantors of commonality in terms of experience with regard to FGM/C. As stated by Hernlund and Shell-Duncan, recent scholarship on FGM/C indicates that restricting studies to one ethnicity to control for context, nationality and type of FGM/C is now becoming “too simplistic a picture” [50]. A plethora of other factors are now coming into play.

## Conclusion

This study sheds light on the specific realities, challenges and successes of women with FGM/C mothering ‘uncut’ daughters. These results have implications for psychosocial workers and mental health practitioners. Professionals should address mothers’ concerns not only in terms of biomedical consequences, but broaden their scope to include psychosocial concerns, notably with regards to mothering in a novel sociocultural context. Women who have left their daughters ‘uncut’ may require information on psychosexual development and counselling on their experience of mothering in a foreign land characterized by a more permissive social context. Practitioners should be sensitive to issues of family dynamics, as pressure from the extended family network can constitute a significant stressor for these mothers. Women who are desirous to forego the practice may require support in facing pressure from the extended family, and support in their attempt to safeguard family bonds while deflecting what remains a cardinal kinship symbol. As a means to promote psychosocial adaptation, professionals may also cultivate positive emotions such as feelings of pride, and bolster self-esteem and empowerment through vicarious protection. In their work with mothers who have undergone FGM/C, mental health professionals should investigate the practice from a semiotic and phenomenological perspective, and explore the reverberations on FGM/C as they bi-directionally impel women’s identity: as mothers, but also as daughters who have undergone this ‘tradition in transition’.

## Abbreviations

FGM/C: Female Genital Mutilation/Cutting
WHO: World Health Organization
